# Neuroplastic Reorganization Induced by Sensory Augmentation for Self-Localization During Locomotion

**DOI:** 10.3389/fnrgo.2021.691993

**Published:** 2021-08-13

**Authors:** Hiroyuki Sakai, Sayako Ueda, Kenichi Ueno, Takatsune Kumada

**Affiliations:** ^1^Human Science Laboratory, Toyota Central R&D Laboratories, Inc., Tokyo, Japan; ^2^TOYOTA Collaboration Center, RIKEN Center for Brain Science, Wako, Japan; ^3^Support Unit for Functional Magnetic Resonance Imaging, RIKEN Center for Brain Science, Wako, Japan; ^4^Graduate School of Informatics, Kyoto University, Kyoto, Japan

**Keywords:** augmentation, plasticity, driving, fMRI, locomotion

## Abstract

Sensory skills can be augmented through training and technological support. This process is underpinned by neural plasticity in the brain. We previously demonstrated that auditory-based sensory augmentation can be used to assist self-localization during locomotion. However, the neural mechanisms underlying this phenomenon remain unclear. Here, by using functional magnetic resonance imaging, we aimed to identify the neuroplastic reorganization induced by sensory augmentation training for self-localization during locomotion. We compared activation in response to auditory cues for self-localization before, the day after, and 1 month after 8 days of sensory augmentation training in a simulated driving environment. Self-localization accuracy improved after sensory augmentation training, compared with the control (normal driving) condition; importantly, sensory augmentation training resulted in auditory responses not only in temporal auditory areas but also in higher-order somatosensory areas extending to the supramarginal gyrus and the parietal operculum. This sensory reorganization had disappeared by 1 month after the end of the training. These results suggest that the use of auditory cues for self-localization during locomotion relies on multimodality in higher-order somatosensory areas, despite substantial evidence that information for self-localization during driving is estimated from visual cues on the proximal part of the road. Our findings imply that the involvement of higher-order somatosensory, rather than visual, areas is crucial for acquiring augmented sensory skills for self-localization during locomotion.

## 1. Introduction

### 1.1. Background

Sensory skills can be augmented through training and technological support. An obvious example is Braille reading, in which well-trained individuals can read letters via tactile sensation when touching Braille symbols. Various devices have been developed to facilitate sensory augmentation. Such devices detect environmental information by using electronic sensors and convert it into stimuli delivered to a sensory organ that is not innately associated with the information. For example, one device translates visual scenes recorded by a digital camera into auditory stimuli by converting elevation to pitch and brightness to loudness (Meijer, 1992). After training with such a device, users are able to discriminate several visual objects without actually seeing them (Striem-Amit et al., 2012). Another research group has developed a waist-belt-type vibration device that constantly displays magnetic north (Nagel et al., 2005). This enables users to utilize a newly acquired magnetic orientation sense to navigate in outdoor environments.

Sensory augmentation is underpinned by neuroplastic reorganization in the brain. A pioneering study by Sadato et al. (1996) demonstrated the activation of visual cortical areas in blind individuals during Braille reading. Similar cortical reorganization in visual cortical areas has been observed after training of blind individuals in distance perception aided by an ultrasound echolocation device (De Volder et al., 1999) or in letter recognition by using an electrotactile stimulation device (Ptito et al., 2005). Such reorganization has also been observed after training of sighted individuals in depth perception by using a device that converts visual scenes into auditory stimuli (Renier et al., 2005), and of both sighted and blind individuals in object recognition with a visual-to-auditory sensory substitution device (Amedi et al., 2007). In the case of this last device, it has been further demonstrated that shape information conveyed by auditory stimuli activates different visual areas, depending on the category of the object (Reich et al., 2011; Striem-Amit et al., 2012; Abboud et al., 2015).

Recently, we proposed a novel sensory augmentation system that assists self-localization during vehicle driving (Ueda et al., 2019). This system translates a vehicle's lateral position in a traffic lane into binaural balance of white-noise loudness, enabling drivers to sense a lane line they are approaching as increased loudness to the ipsilateral ear and decreased loudness to the contralateral ear. By using this auditory-based self-localization assistance system for locomotion, we demonstrated in a simulated driving environment that drivers developed the ability to control the vehicle accurately by using auditory cues, even when the visual information needed to estimate vehicle lateral position (i.e., the proximal part of the road) was occluded. This was the first successful attempt to show the applicability of sensory augmentation to time-sensitive daily-life situations in healthy individuals. However, the underlying neural mechanisms of the training effects remain unclear.

### 1.2. Objective

Here, by using functional magnetic resonance imaging (fMRI), we aimed to identify the neuroplastic reorganization induced by sensory augmentation training for self-localization during locomotion. Specifically, we employed a pretest-training-posttest design comprising three separate fMRI sessions (pretest, posttest, and follow-up test), before and after driver training with or without the sensory augmentation assistance system in a simulated environment. In the pretest fMRI session, activation of the response to auditory stimuli conveying vehicle lateral position information was investigated by using a conventional block design protocol. After the pretest fMRI session, participants were randomly assigned to one of two training conditions (normal driving [ND] or sensory augmentation [SA] conditions) and accordingly performed 8 days of driver training. On the day after the last training day, a posttest fMRI session was conducted by using a protocol identical to that applied in the pretest. The fMRI session was further repeated approximately 1 month later as a follow-up test. We then identified training-related changes in auditory responses under each condition.

Previous studies examining neuroplasticity after sensory augmentation have reported a clear tendency for trained sensory information to become processed in the brain areas for a sensory modality associated with the content of the information, rather than the carrier of the information. Therefore, we expected that vehicle lateral position translated into auditory stimuli would be processed in visual areas after training, because vehicle lateral position is considered to be estimated from the visual cues contained in driving scenes as viewed from the driver's perspective (Land and Horwood, 1995; Billington et al., 2010; Frissen and Mars, 2014).

## 2. Materials and Methods

### 2.1. Participants

Fourteen adults (2 females; 12 males) aged 20–28 years participated in the study and received financial compensation for their participation. All participants self-reported normal or corrected-to-normal vision and normal hearing. In addition, all participants were free from psychiatric, neurological, and major medical illnesses, as determined by medical history. They were all right-handed according to the Edinburgh Handedness Inventory (Oldfield, 1971). Each participant provided written informed consent. Experimental protocols were approved by the RIKEN Research Ethics Committee [Wako3 28-17(4)] and were conducted according to the principles of the Declaration of Helsinki.

### 2.2. Study Design

To identify neuroplastic reorganization associated with sensory augmentation for self-localization during locomotion, we employed a pretest-training-posttest design comprising three separate fMRI sessions (pretest, posttest, and follow-up). After the pretest fMRI scan, participants were randomly assigned to either the ND or SA condition, and they accordingly performed eight sequential days of training (excluding weekends and occasional absences) in a simulated environment. On the day after the last training day, a posttest fMRI scan was conducted. In addition, another follow-up fMRI scan was performed ~1 month after the end of the training. To minimize observer effects, the experimenters conducting the fMRI sessions were blind to which participant was assigned to which training condition.

### 2.3. Driving Training

**Figure 1:**
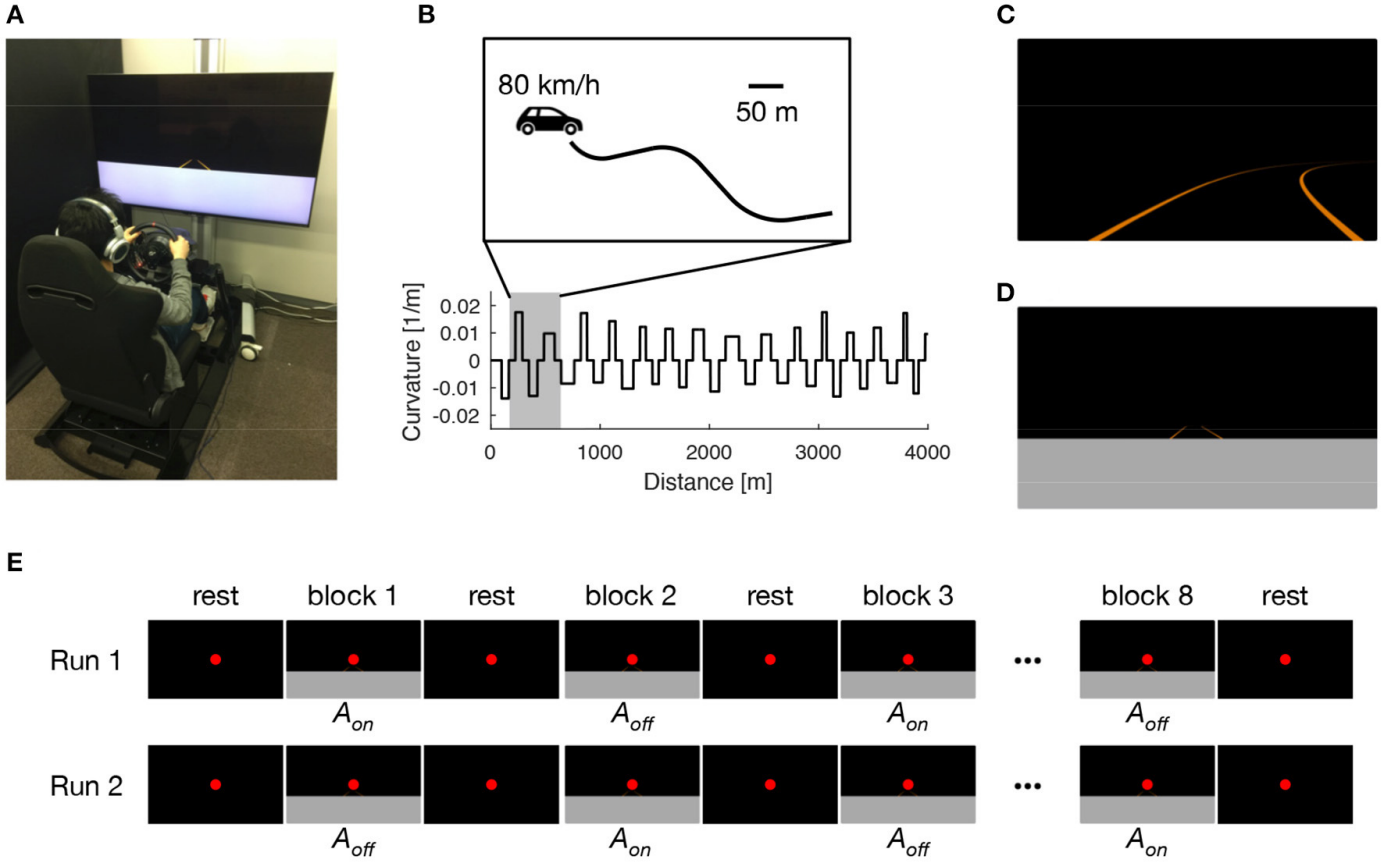
Experimental setup. Participants performed 8 days of lane-keeping training on a custom-made driving simulator **(A)**. Driving courses consisted of a winding road that alternately curved leftward and rightward with a random curvature between 1/120 and 1/60 m^−1^, interleaved with straight sections with a constant length of 60 m **(B)**. Under normal driving (ND) condition, driving scenes were presented with auditory white noise with a constant loudness to both ears via headphones **(C)**. Under sensory augmentation (SA) condition, the lower part of the driving scene was occluded to restrict the visual cues needed for estimating vehicle lateral position, and information regarding vehicle lateral position was instead provided via binaural auditory stimuli **(D)**. The pretest and posttest MRI sessions comprised two block-design runs, in which participants were exposed to sensory stimuli that were relevant to the lane-keeping task under SA condition. In each run, driving scenes were presented alternately with (*A*_*on*_) and without (*A*_*off*_) auditory cues for vehicle lateral position **(E)**.

For sensory augmentation training, we used a custom-made driving simulator comprising a fixed-base cockpit (GTD-SPECi, Rossomodello Co., Ltd., Tomioka, Japan), a force-feedback steering device (T500RS, Guillemot Corp., Carentoir, France), and a 60-inch LCD monitor (LC60XL10, Sharp Corp., Sakai, Japan) located in front of the cockpit (Figure 1A). The system was identical to the one used in our previous behavioral study (Ueda et al., 2019). Driving scenes as viewed from the driver's perspective were reconstructed at 60 Hz and displayed on the monitor, subtending horizontal and vertical visual angles of 66° and 43°, respectively, at a viewing distance of 105 cm.

On each of the 8 training days, participants performed 20 trials of a lane-keeping task (180 s for each trial) under their assigned training condition (i.e., ND or SA condition). In each trial, participants were required to keep their vehicle in the center of a traffic lane by using the steering wheel. The traffic lane was defined by left and right lane lines, giving a lane width of 3.5 m. The lane alternately curved leftward and rightward with a random curvature between 1/120 and 1/60 m^−1^; this was interleaved with straight sections with a constant length of 60 m (Figure 1B). The vehicle automatically traveled with a constant speed of 80 km/h and, therefore, no pedal operations were needed. No other road users were present throughout the task. Under ND condition (Figure 1C), white noise with a constant loudness (−50 dB attenuated from the maximum level that we predetermined to be comfortably tolerable to participants) was presented to both ears of participants via headphones during the entire lane-keeping task. In contrast, under SA condition (Figure 1D), the lower part of the driving scenes (i.e., the proximal part of the road) was occluded to restrict the visual cues needed to estimate vehicle lateral position. Instead, information regarding vehicle lateral position was provided via binaural auditory stimuli. Specifically, leftward (rightward) deviation of vehicle lateral position from the center of the lane was signaled by increasing the loudness of white noise in the left (right) ear and decreasing the loudness of white noise in the right (left) ear with a constant gain of 25 dB/m; in the center of the lane, the loudness level was equal in both ears (−50 dB). Participants assigned to SA condition were instructed in advance regarding the meaning of the auditory stimuli. Under both conditions, the entire experiment, including preparation and rest breaks (<1 min) between trials, took <90 min on each training day.

Driving performance in the lane-keeping task was evaluated in terms of accuracy and smoothness of vehicle control by using the standard deviation of vehicle lateral position (SDLP) and the maximum steering wheel velocity during curve negotiation (SWV), respectively. There is accumulating evidence that SDLP reflects compensatory steering control that makes use of the visual information provided by the proximal part of the road, whereas SWV reflects anticipatory steering control that makes use of the information provided by the more distant part of the road (Frissen and Mars, 2014; Ueda et al., 2019). For both SDLP and SWV, lower values represent better performance. To examine the effects of the training on driving performance, we estimated the learning plateau and learning rate under each training condition. First, we computed the trajectory of each performance metric (i.e., SDLP or SWV) as a function of trial number (total, 160 trials) for each participant and fitted an exponential function [*Y* = *a*+*b*exp(−*cX*) where *Y* is a performance metric, *X* is the trial number, and *a*, *b*, and *c* are regression parameters] to the mean trajectory averaged across participants to estimate the learning plateau (*a*) and learning rate (*c*) with a 95% confidence interval (CI).

### 2.4. MRI Data Acquisition

MRI data acquisition was performed on a Siemens 3T Prisma scanner with a 64-channel head array coil (Siemens Medical System, Erlangen, Germany). In each fMRI session, a high-resolution T1-weighted structural image was acquired by using a 3D MPRAGE sequence (Mugler and Brookeman, 1990) with an echo time (TE) of 3.25 ms, a repetition time (TR) of 1,700 ms, an inversion time (TI) of 0.9 s, a flip angle (FA) of 8°, a field of view (FOV) of 256 × 256 mm^2^, a matrix size of 256 × 256, a slice thickness of 1 mm, 192 contiguous sagittal slices, and an acceleration factor of 2 for the GRAPPA parallel imaging technique (Griswold et al., 2002). Then, fMRI data were collected by using a gradient echo T2^*^-weighted echo-planar imaging (EPI) sequence with a TE of 30 ms, a TR of 1,556 ms, an FA of 74°, an FOV of 200 × 200 mm^2^, a matrix size of 100 × 100, a slice thickness of 2 mm, 72 contiguous axial slices, an acceleration factor of 2 for the GRAPPA parallel imaging technique, and a multi-band factor of 3.

During fMRI, participants were exposed to sensory stimuli that were relevant to the lane-keeping task under SA condition (Figure 1E). Specifically, after a 16-s rest period, participants were given a 16-s stimulation period followed by a 16-s rest period 8 times (giving a “run” of 272 s in total, corresponding to 175 EPI volumes). For the stimulation periods, driving video clips were created from driving log data in which an experimenter (HS) had performed lane-keeping under SA training condition. The video clips were displayed on a translucent screen with visual angles of 26.3 and 15.6°. In half of the video clips in each run, the audio tracks containing auditory cues for vehicle lateral position were present (*A*_*on*_). In the other half, the audio tracks were removed (*A*_*off*_). These two kinds of video clips were presented alternately in a run. Throughout the run, regardless of whether the participant was in a rest period or a stimulation period, a red dot was presented constantly for fixation. The run was presented twice to each participant, with the first run starting with *A*_*on*_ after the first rest period and the second starting with *A*_*off*_ instead. In each run, participants were asked to answer which lane line (left or right) was being approached by pressing either the left or right button of a hand-held switch box with their right index finger or middle finger, respectively. The purpose of this instruction was to maintain the participant's attention on the sensory stimuli, rather than to evaluate performance in the scanner. Throughout the entire fMRI session, we confirmed that participants were in a state of arousal by monitoring their eyes via a camera. In addition, their respiratory and cardiac signals were collected by using a pressure sensor and a pulse oximeter, respectively. These electrophysiological signals were used afterwards to remove physiological fluctuations from the EPI images (Hu et al., 1995).

### 2.5. fMRI Data Analysis

Task fMRI data were preprocessed for each participant using SPM12 (v7487; www.fil.ion.ucl.ac.uk/spm) with the CAT12 toolbox (r1184; www.neuro.uni-jena.de/cat). First, to obtain a deformation field for accurate normalization of EPI images, a T1-weighted structural image collected in the pretest session was processed by using a segmentation procedure in CAT12. Second, EPI images were preprocessed by using SPM12. All EPI images acquired in the three fMRI sessions were realigned to the first image in the pretest session for head motion correction and then coregistered to the structural image in the pretest session. The coregistered EPI images were normalized to MNI (Montreal Neurological Institute) space by using the deformation field and then smoothed with an isotropic Gaussian kernel with a full width at half maximum of 6 mm.

To identify neuroplastic reorganization induced by the sensory augmentation training, we performed a voxelwise general linear model analysis. At the first level, we modeled the two stimulus effects (*A*_*on*_ and *A*_*off*_) for each run by using boxcar functions convolved with the canonical hemodynamic response function. By contrasting the two stimulus conditions (*A*_*on*_>*A*_*off*_) for each fMRI session, we obtained activation in response to auditory cues for vehicle lateral position. At the second level, we entered these individual contrast images into a 2-by-3 full factorial model with a between-subjects factor of training condition (ND, SA) and a within-subjects factor of fMRI session (pretest, posttest, follow-up). We then performed a conjunction analysis of auditory responses in all fMRI sessions (pretest ∩ posttest ∩ follow-up) to identify the brain regions consistently activated before and after the lane-keeping training. We also compared auditory responses between different fMRI sessions to identify brain regions that were more active after training, compared with before training (i.e., pretest < posttest and pretest < follow-up). For all analyses, the results were considered statistically significant at *P * < 0.01 cluster-level family-wise error (FWE) corrected for multiple comparisons, with a voxel-level threshold of *P* < 0.005 uncorrected.

## 3. Results

### 3.1. Driving Training

**Figure 2:**
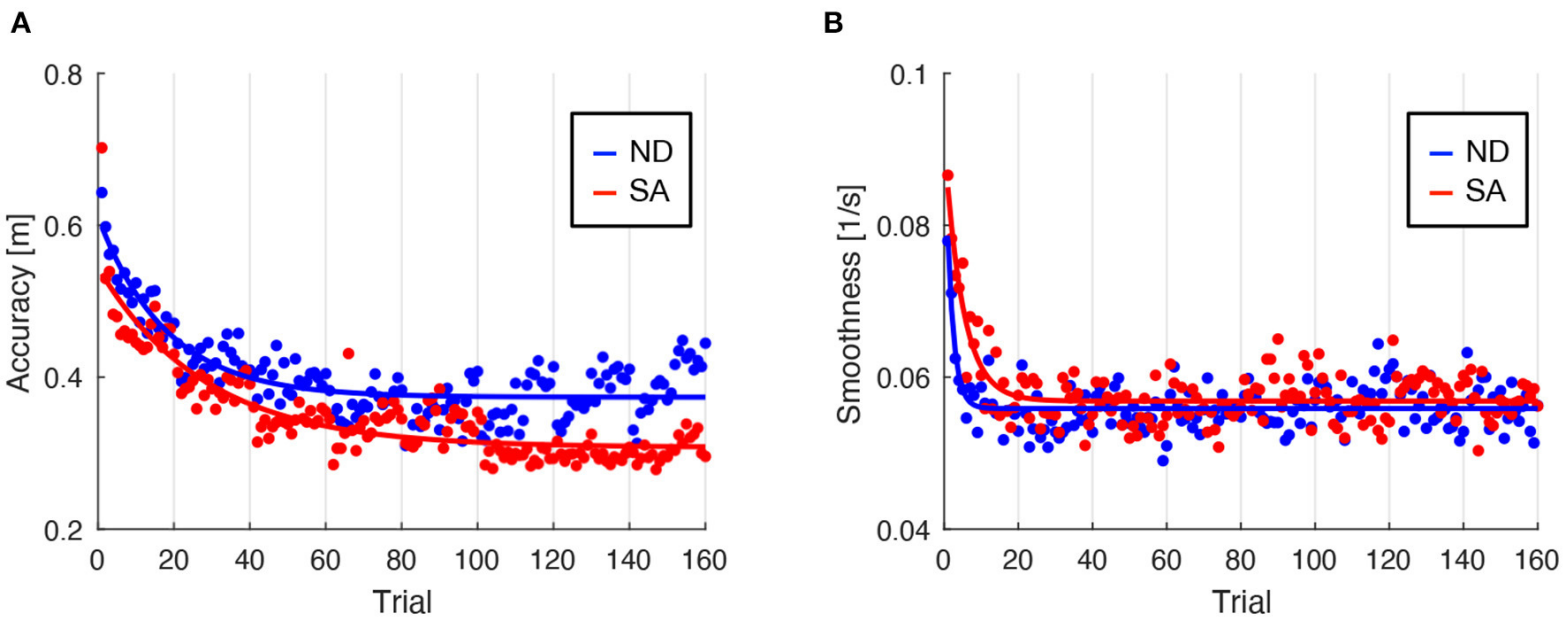
Trajectories of driving performance during lane-keeping training. Driving performance was indexed with regard to the accuracy **(A)** and smoothness **(B)** of steering as a function of trial number. Blue and red dots represent mean performance in each trial, separately averaged across participants under ND and SA conditions. Each line denotes a learning curve fitted to determine learning rate and learning plateau. Vertical thin lines indicate respective training days.

Under both ND and SA conditions, lane-keeping accuracy as assessed by using SDLP improved as training progressed (Figure 2A). Regression analysis revealed that learning rate for lane-keeping accuracy was slower under SA condition (0.036; 95% CI, 0.29–0.42) than under ND condition (0.055; 95% CI, 0.044–0.067), and that the learning plateau was lower under SA condition (0.31; 95% CI, 0.30–0.32) than under ND condition (0.37; 95% CI, 0.37–0.38). Lane-keeping smoothness, as assessed by using SWV, also improved as training progressed, under both conditions (Figure 2B). Regression analysis revealed that learning rate for lane-keeping smoothness was slower under SA condition (0.20; 95% CI, 0.15–0.25) than under ND condition (0.55; 95% CI, 0.32–0.78) and that the learning plateaus were comparable under SA (0.057; 95% CI, 0.056–0.057) and ND (0.056; 95% CI, 0.055–0.056) conditions. Overall, lane-keeping performance under SA condition improved more slowly during training, but after training it was eventually comparable to, or better than, that under ND condition, even though for SA participants the visual information essential for lane-keeping was unavailable.

### 3.2. Brain Activation

During fMRI, participants kept their eyes open except for natural blinking. In addition, all participants pressed the buttons more than 3 times in every task block (Supplementary Figure 1). These results suggest that the participants were engaged in the task.

Conjunction analysis of auditory responses across the three fMRI sessions (i.e., pretest, posttest, and follow-up) revealed significant brain activation in the superior temporal gyri bilaterally under both ND and SA conditions (Table 1), although the left clusters did not satisfy the statistical criteria under ND condition. These results indicate that auditory cues for vehicle lateral position consistently activated temporal auditory areas.

**Table 1:** Brain regions activated in response to auditory cues for self-localization.

**Region**	***x***	***y***	***z***	***t***	**Size**
(ND, pretest ∩ posttest ∩ follow-up)					
Superior temporal gyrus	50	−28	12	5.95	511
Superior temporal gyrus	−34	−36	16	4.05	111^†^
Superior temporal gyrus	−56	−36	12	3.49	79^†^
(SA, pretest ∩ posttest ∩ follow-up)					
Superior temporal gyrus	66	−32	12	7.09	824
Superior temporal gyrus	−62	−38	10	6.48	806
(ND, pretest < posttest)					
Pre-supplementary motor area	10	12	56	5.76	1284
Anterior insular cortex	−36	8	6	5.63	392
(SA, pretest < posttest)					
Parietal operculum	−60	−28	18	3.91	372
Supramarginal gyrus	−58	−28	28	3.73	
Postcentral gyrus	−56	−28	52	3.60	
Postcentral gyrus	64	−16	22	3.76	392
Supramarginal gyrus	66	−24	24	3.62	

*Statistical significance was set at cluster-level P <0.01, family-wise error (FWE) corrected for multiple comparisons; the clusters marked with a dagger (*

† *) were not large enough to satisfy this criterion. ND and SA represent normal driving and sensory augmentation training conditions, respectively. Cluster locations are given in Montreal Neurological Institute coordinates*.

**Figure 3:**
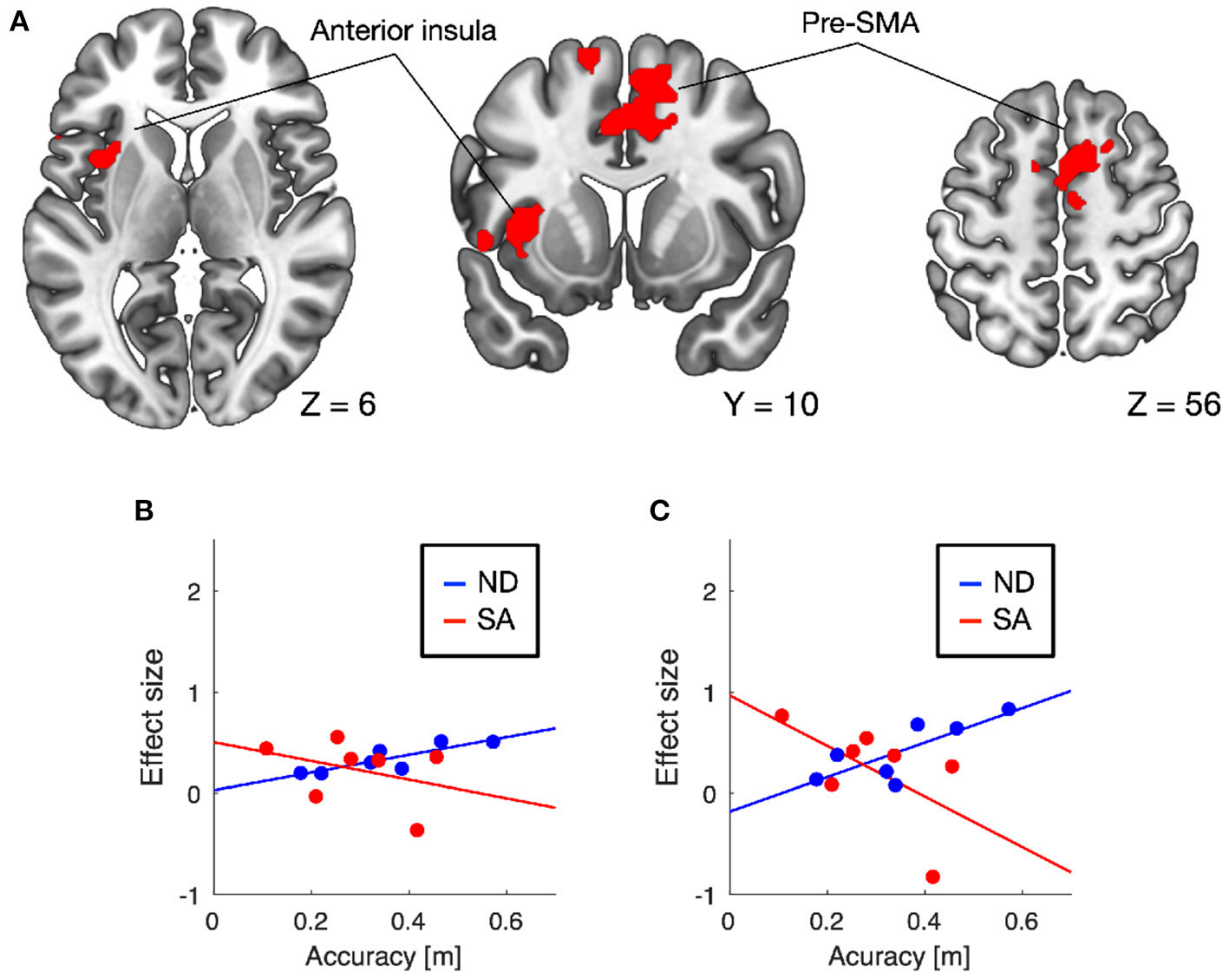
Brain regions showing increased auditory responses after lane-keeping training under ND condition (pretest < posttest). Significant clusters (family-wise error (FWE) corrected *P* < 0.01) were found in the pre-supplementary motor area (pre-SMA) and left anterior insula **(A)**. In both clusters, increased activation after the training was positively correlated with lane-keeping accuracy under ND condition, but negatively under SA condition (**B**, pre-SMA; **C**, anterior insula).

**Figure 4:**
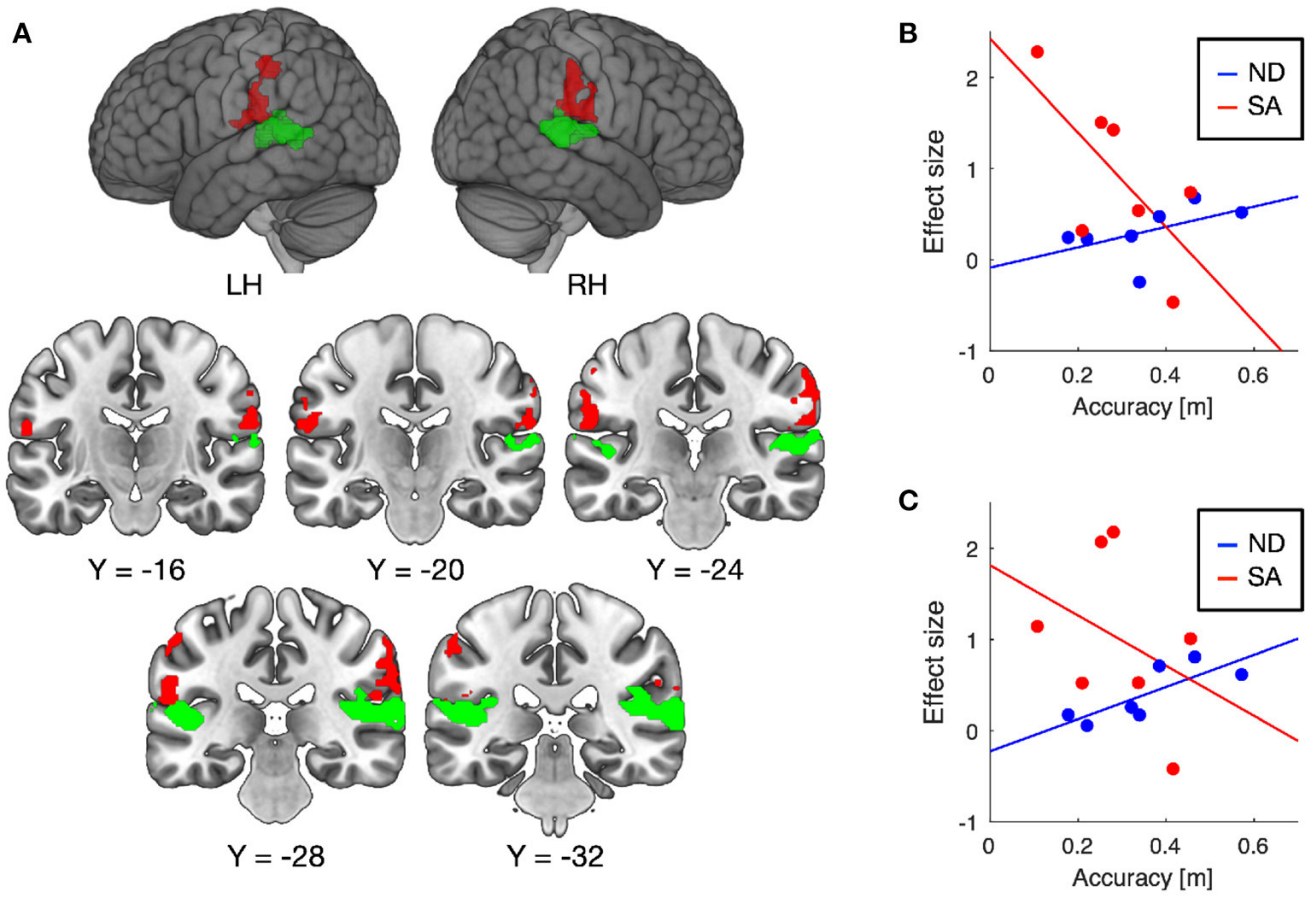
Brain regions showing increased auditory responses after lane-keeping training under SA condition (pretest < posttest) **(A)**. Significant clusters (FWE-corrected *P* < 0.01) were found in the somatosensory areas bilaterally (red), which were not overlapped with the superior temporal auditory areas (green) revealed by the conjunction analysis (i.e., pretest ∩ posttest ∩ follow-up). In both clusters, increased activation after the training was negatively correlated with lane-keeping accuracy under SA condition, but positively under ND condition (**B**, left cluster; **C**, right cluster).

Training-induced changes (pretest < posttest) in auditory responses differed between SA and ND conditions (Table 1). Under ND condition, comparison of pretest and posttest auditory responses revealed increased activation in the pre-supplementary motor area (pre-SMA) and anterior insular cortex (Figure 3A). In contrast, under SA condition, increased activation in the posttest fMRI compared with the pretest fMRI was found in the somatosensory areas bilaterally, including in the parietal operculum, supramarginal gyrus (SMG), and postcentral gyrus (Figure 4A), which were adjacent to, but not overlapping with, the superior temporal auditory areas revealed by the conjunction analysis. Under both conditions, no significant clusters were found in the follow-up fMRI compared with the pretest fMRI. These changes in the posttest fMRI results can be regarded as the signatures of neuroplastic reorganization induced by the lane-keeping training.

Furthermore, we examined the relationships between training-induced neuroplastic changes (pretest < posttest) and driving performance achieved by the training (i.e., the learning plateau in lane-keeping accuracy; Figure 2A). In the somatosensory areas, increased activation after the training was negatively correlated with lane-keeping accuracy under SA condition (left cluster, *r* = −0.69, *P* = 0.044; right cluster, *r* = −0.36, *P* = 0.21; one-tailed *t*-test) but positively correlated under ND condition (left cluster, *r* = 0.51, *P* = 0.12; right cluster, *r* = 0.79, *P* = 0.017; Figures 4B,C), suggesting that better lane-keeping performance under SA condition was associated with greater involvement of the somatosensory areas in auditory processing after the training. A similar tendency was also found in both the pre-SMA (ND, *r* = 0.86, *P* = 0.0069; SA, *r* = −0.35, *P* = 0.22; Figure 3B) and the anterior insular cortex (ND, *r* = 0.78, *P* = 0.019; SA, *r* = −0.59, *P* = 0.083; Figure 3C), suggesting that better lane-keeping performance under ND condition was associated with less involvement of these frontal areas in auditory processing after the training. In all clusters, between-group differences in correlation coefficients were statistically significant (for the left somatosensory cluster, *P* = 0.023; for the right somatosensory cluster, *P* = 0.020; for the pre-SMA cluster, *P* = 0.010; for the anterior insular cluster, *P* = 0.0073; one-tailed *Z*-test).

## 4. Discussion

Increasing attention has been paid to sensory augmentation for not only sensory impaired but also healthy individuals, because it can open new horizons for human-machine/computer interface development by reconsidering human-environment interactions (Di Pino et al., 2014). We previously demonstrated the potential of an auditory-based self-localization assistance system for locomotion in a simulated driving environment; this demonstration pioneered the application of sensory augmentation to time-sensitive daily-life situations in healthy individuals (Ueda et al., 2019). However, the neural mechanisms underlying sensory augmentation for self-localization during locomotion remain unclear. Identifying the neural underpinnings of augmentation could provide information that would improve our self-localization assistance systems and facilitate the development of novel sensory augmentation devices to assist locomotion.

Here, we measured brain activation in response to auditory cues on vehicle lateral position before and after lane-keeping training to identify training-induced neuroplastic reorganization. Although we expected auditory responses in the occipital visual areas after the training, we found training-induced increases in the activation of somatosensory areas in the parietal lobe. We also found that greater involvement of somatosensory areas in auditory processing after the training was associated with better lane-keeping performance under SA condition. Not only the parietal operculum, which is considered to be the location of the secondary somatosensory cortex (Ruben et al., 2001; Eickhoff et al., 2006), but also the SMG is involved in somatosensory processing as a human homologue of the tertiary somatosensory cortex in the monkey (Caselli, 1993; Hagen and Pardo, 2002). Our findings appear to be consistent with the neuroplastic changes involved in auditory-induced somatosensory sensation after stroke. Beauchamp and Ro (2008) investigated auditory responses in a stroke patient complaining of sound-touch synesthesia and found substantial activation within the secondary somatosensory cortex in response to auditory stimuli. Furthermore, there is growing evidence of the inherent multimodal nature of the higher-order somatosensory cortices. Bremmer et al. (2001) demonstrated that the SMG was activated consistently by visual, auditory, and tactile motion stimuli. More recently, Pérez-Bellido et al. (2018) reported that auditory frequency information was represented in somatosensory areas, including the SMG, as well as in the auditory cortices. These findings clearly indicate that auditory information can be processed in higher-order somatosensory cortices. Taken together, our data suggest that the use of auditory cues for self-localization during locomotion relies on multimodality in higher-order somatosensory cortices rather than the occipital visual cortices, even though the vehicle lateral position conveyed by the auditory cues in this study is usually estimated from visual cues within the proximal part of the road (Land and Horwood, 1995; Frissen and Mars, 2014; Ueda et al., 2019).

However, another interpretation is possible because there were differences in visual as well as auditory stimuli during driving training between ND and SA conditions. Specifically, the lower part of the driving scenes (i.e., the proximal part of the road) was visually occluded in SA, but not ND, condition. Therefore, the results could be interpreted as indicating that improved driving performance in SA condition resulted from the completion of occluded visual information rather than the complementary use of auditory cues (sensory augmentation). Although this interpretation cannot be ruled out in the current experiment, our previous behavioral study (Ueda et al., 2019) revealed that when the proximal part of the road is occluded, driving performance does not improve without auditory cues for self-localization. Thus, we concluded that it is more likely that somatosensory involvement in auditory processing after SA training resulted from the acquisition of augmented sensory skill using auditory cues for self-localization rather than visual completion.

It could be argued that this sensory augmentation for self-localization during locomotion resembles echolocation and therefore that they are likely to share neural substrates. Echolocation is an augmented sensory skill by which the sound reverberation of mouth clicks is used to infer spatial information in the surrounding environment, and it can be used for navigation by blind people. From a functional point of view, echolocation and the sensory augmentation that we examined here can both be regarded as auditory-based localization skills. However, their neural bases appear to differ from each other. Several lines of evidence demonstrate the involvement of occipital visual, rather than somatosensory, areas in echolocation (Thaler et al., 2011, 2014; Wallmeier et al., 2015). Furthermore, Thaler and Foresteire (2017) reported that, in sighted individuals, echolocation performance was disrupted by task-unrelated visual, but not tactile, stimuli, also suggesting that there is a lack of involvement of the somatosensory areas in echolocation. The major difference between our findings and those of these previous echolocation studies is in the action demanded for locomotion. Our participants were required to control a vehicle by using augmented sensory information for self-localization, whereas in the previous studies participants were asked to extract spatial information on the surrounding objects from echoes. The use of augmented sensory information for self-localization during locomotion might be crucial for the involvement of somatosensory areas. In fact, one previous study reported the echo-related activation of extensive parietal areas in a situation where the use of echolocation ability to detect path directions during walking was required (Fiehler et al., 2015). The neuroplastic reorganization induced by sensory augmentation may depend not only on what kinds of content are conveyed via an augmented sense, but also on how the augmented sensory information is used to accomplish task demands. This notion requires further clarification in future research.

Another important finding of our study was that superior lane-keeping accuracy was achieved with, rather than without, the auditory-based self-localization assistance system. It is evident that the visual information contained in the proximal part of the road is typically critical to accurate lane-keeping (Land and Horwood, 1995; Frissen and Mars, 2014). By using the same experimental setup, we previously demonstrated that when the proximal part of the road was occluded, lane-keeping accuracy was markedly degraded and did not improve with training (Ueda et al., 2019). However, we observed that the learning curve of lane-keeping accuracy reached a better plateau under SA condition than under ND condition. This result may be attributable to more accurate and precise feedback of lane-keeping errors under SA condition. In general, sensorimotor learning tasks require sensory error signals if a person is to achieve fine motor control (Kawato et al., 1987). Under ND condition and in typical visual-based vehicle driving, drivers are required to estimate the vehicle lateral position by using visual cues from the proximal part of the road. In contrast, the auditory cues used under SA condition can provide exact information about the vehicle lateral position. Nevertheless, in terms of both accuracy and smoothness performance metrics, learning was slower under SA than under ND condition. This presumably reflects the additional cognitive cost of utilizing auditory cues for self-localization.

We also found training-induced neuroplastic changes under ND condition. This was an unexpected result, because under ND condition the auditory stimuli presented during lane-keeping training were totally irrelevant to the task. A possible interpretation for this is that the cognitive control required to ignore the task-irrelevant auditory stimuli during lane-keeping training resulted in training-induced greater activation of the pre-SMA and the anterior insular cortex. In fact, both these regions are considered part of the cognitive control network (Cole and Schneider, 2007; Niendam et al., 2012). In addition, Smucny et al. (2013) showed that the pre-SMA is engaged when auditory distraction is present during a highly demanding cognitive task. The anterior insular cortex is also known to play a crucial role in suppressing distractor inference (Bunge et al., 2002). This interpretation seems to dovetail with lesser involvement of the superior temporal auditory cortex under ND condition than under SA condition (Table 1). Under this interpretation, furthermore, more involvement of the abovementioned frontal regions under ND condition would indicate the assignment of more cognitive resources to distractor inference suppression; this is consistent with our finding that under ND condition, lane-keeping performance was negatively associated with increased involvement of these areas after the training.

Several limitations of our study should be noted. First, the small sample size restricts the ability to generalize our findings. In particular, we identified training-induced neuroplastic changes only as within-group differences, not as between-group differences (i.e., interaction between training condition and fMRI session). Replication with a larger sample size is needed to improve the generalizability of our results. Second, brain activation associated with sensory augmentation for self-localization needs to be investigated by using a more realistic driving environment. We made a substantial effort to expose participants in the MRI scanner to sensory stimuli similar to those experienced during sensory augmentation training. However, the sensory stimulation remained different between inside and outside the scanner (e.g., the size of the visual stimuli). In addition, we examined brain activation during passive exposure to sensory stimuli, but not during active behaviors using augmented sensory information. According to previous neuroimaging studies (Uchiyama et al., 2003, 2012), active compared with passive driving additionally activates not only motor areas but also various sensory and association areas. It will be important for future studies to investigate the role of somatosensory responses to auditory cues for self-localization in such driving-related networks. Because there is empirical evidence that brain activation associated with sensory augmentation is context-dependent (Sadato et al., 1996), differences in stimuli and behavior might have influenced our findings.

## 5. Conclusion

We demonstrated here that sensory augmentation for self-localization during locomotion results in extensive neuroplastic reorganization in the human cerebral cortex. Interestingly, although we expected training-induced reorganization in the occipital visual areas, we observed neuroplastic changes in higher-order somatosensory areas. This finding suggests that the involvement of somatosensory, rather than visual, areas is crucial for acquiring augmented sensory skills for self-localization during locomotion, even though self-localization is considered to rely heavily on vision. Our data also showed that, depending on how it is used, sensory augmentation can enable better performance in healthy individuals, particularly in situations where the information provided by an augmented sense initially requires the complex computation of sensory signals. Our findings will facilitate further applications of sensory augmentation to human-computer/machine interface development.

## Data Availability Statement

The raw data supporting the conclusions of this article will be made available by the authors, without undue reservation.

## Ethics Statement

The studies involving human participants were reviewed and approved by RIKEN Research Ethics Committee. The patients/participants provided their written informed consent to participate in this study.

## Author Contributions

HS, SU, and TK contributed to conception and design of the study. SU and KU performed the experiment. HS and SU performed the data analysis and wrote the first draft of the manuscript. All authors contributed to manuscript revision, read, and approved the submitted version.

## Conflict of Interest

The authors declare that this study received funding from Toyota Motor Corporation. The funder was not involved in the study design, collection, analysis, interpretation of data, the writing of this article or the decision to submit it for publication. In addition, HS was employed by Toyota Central R&D Laboratories. The remaining authors declare that the research was conducted in the absence of any commercial or financial relationships that could be construed as a potential conflict of interest.

## Publisher's Note

All claims expressed in this article are solely those of the authors and do not necessarily represent those of their affiliated organizations, or those of the publisher, the editors and the reviewers. Any product that may be evaluated in this article, or claim that may be made by its manufacturer, is not guaranteed or endorsed by the publisher.
