# Supplementary material for: Neuroplastic Reorganization Induced by Sensory Augmentation for Self-Localization During Locomotion
Source: Front Neuroergon. 2021 Aug 13;2:691993. doi: 10.3389/fnrgo.2021.691993 (PMC10790880; doi:10.3389/fnrgo.2021.691993)
Supplement: Supplementary file 1 [file Image_1.pdf]

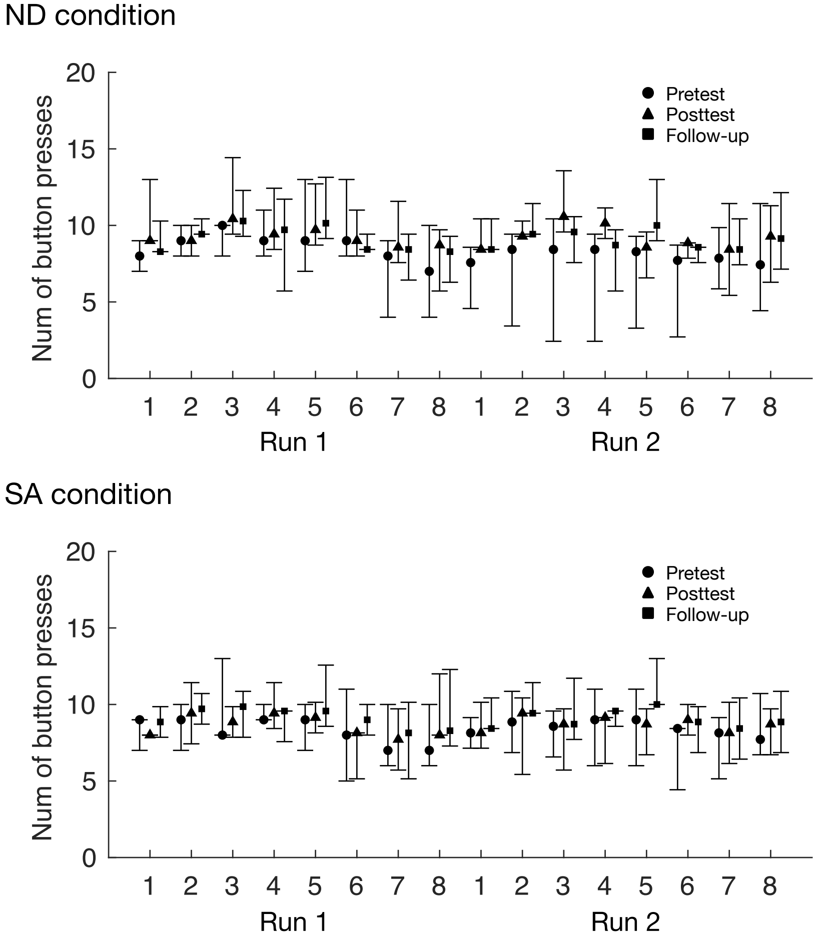

Figure S1. The number of button presses as a function of task block. Symbols and error bars denote the median and min/max values in each task block. ND and SA refer to normal driving and sensory augmentation conditions, respectively.
